# Encapsulating volatiles by spray drying: The choice of dextrose equivalent influences d-limonene retention via skin formation and particle morphology

**DOI:** 10.1016/j.crfs.2025.101224

**Published:** 2025-10-10

**Authors:** Ana K.P. Jauhari, Viktorija Lucenko, Meinou N. Corstens, Patrick F.C. Wilms, Maarten A.I. Schutyser

**Affiliations:** Laboratory of Food Process Engineering, Wageningen University & Research, P.O. Box 17, 6700 AA, Wageningen, the Netherlands

**Keywords:** Pea protein isolate, Encapsulation efficiency, Skin formation, Surface oil, Essential oils, Droplet drying

## Abstract

Maltodextrin is extensively used as a wall material for volatile encapsulation via spray drying. The dextrose equivalent (DE) is hypothesized to influence the retention of volatiles by affecting the rate of skin formation. However, the DE is also known to affect final particle morphology, and it is not known whether skin formation rate or particle morphology has the most significant influence on retention. This study investigates the encapsulation of d-limonene (diluted in sunflower oil) by emulsification and subsequent spray drying. It focused on understanding the impact of the DE value of maltodextrin on the d-limonene retention after spray drying by evaluating the resulting powder properties and particle morphology. Four formulations were prepared by varying the DE value of maltodextrin (DE6, 12, 21, and 38) using the same drying conditions and having a similar concentration of oil (6 wt%), pea protein isolate (0.9 wt%), and solids (20 wt%). Results showed that the DE value influences d-limonene retention due to differences in the formation of surface oil that is affected by the morphology of the particle formed during drying. The powder containing DE6 resulted in the highest surface oil percentage (6.6 ± 0.8 %) with the lowest d-limonene retention (57.6 ± 2.4 %). These results were attributed mainly to the formation of holes and large cavities in the particle as the droplet skin formed and dehydrated. This phenomenon created additional surface area that allowed for increased d-limonene losses, thereby challenging the initial hypothesis that lower-DE maltodextrin induces rapid skin formation and thereby improves volatile retention. Instead, the findings of this study emphasize that controlling the morphology and structural integrity of spray-dried emulsions is crucial for improving the retention of volatile compounds like d-limonene.

## Highlights

• Volatiles are released more from encapsulated oil than from surface oil.
• The particle morphology during drying influences the volatile retention.
• Higher maltodextrin DE values result in increased volatile retention.
• Lower maltodextrin DE values lead to reduced oil droplet stability during drying.
• There is a trade-off between rapid skin development and cavity formation.

## Graphical abstract

**Figure:**
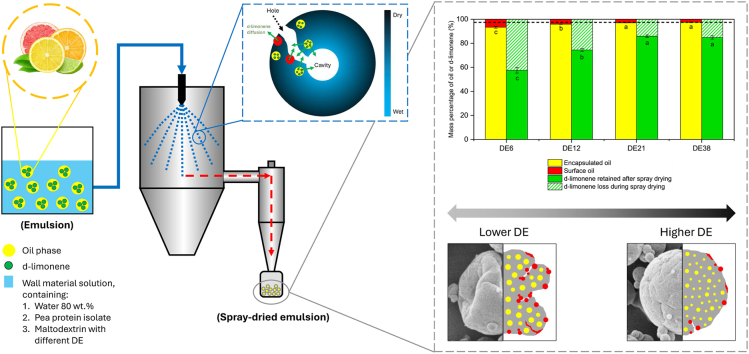

## 1 Introduction

Essential oils are plant-based oils that contain various volatile compounds responsible for distinctive aromas and, in some cases, offer health benefits (Miyazato, 2018). Due to these properties, they are widely applied in various industries, especially the food sector, where they play a significant role in shaping consumer perception. Nonetheless, the high volatility and susceptibility to oxidation of these oils present challenges for preserving their stability during food processing. Spray drying presents an effective strategy to encapsulate essential oils, protecting them within a matrix to enhance their shelf life and preserve their characteristics (Paramita et al., 2012). Nevertheless, this process is challenging, as volatiles are still partially lost during spray drying (Peng et al., 2023; Takashige et al., 2020). To encapsulate essential oils, they should be emulsified in an aqueous phase containing an emulsifier and soluble wall material before being subjected to spray drying. The choice of wall material is crucial as it influences the encapsulation efficiency and properties of the final powder. Maltodextrin is often used as the wall material and its effectiveness is largely determined by its Dextrose Equivalent (DE) value, which indicates the degree of hydrolysis and molecular weight. Maltodextrin with a lower DE value has a lower degree of hydrolysis and thus a higher molecular weight and, consequently, a higher glass transition temperature (T_g_) (Siemons et al., 2020). The DE value affects the viscosity and morphology of the spray-dried emulsions. Typically, formulations with lower DE values exhibit higher viscosities at similar dry matter content and show more elastic behavior, which leads to earlier skin formation, larger vacuoles, and increased porosity upon spray drying (Both et al., 2019). It is also noted that Li et al. (2020) observed that changes in volatile retention for different DE values of maltodextrin may be attributed to interactions between volatiles and maltodextrin, with increasing interactions for higher DE values of maltodextrin leading to more volatile retention. Several studies have specifically investigated the encapsulation of *d*-limonene by spray drying and confirmed the strong influence of wall material composition and its properties on retention after the spray drying process. For instance, Burgos-Díaz et al. (2024) reported that pickering emulsions stabilized with agro-food byproducts improved d-limonene retention and oxidative stability compared to conventional systems, while Takashige and Watanabe (2025) showed that retention decreased with higher loading ratios of d-limonene in maltodextrin matrices due to increased surface oil formation. More recently, Jauhari et al. (2025) emphasized the role of emulsion droplet size distribution during atomization in determining d-limonene retention in spray-dried powders. In addition, Kurtz et al. (2025) also investigated the influence of different molecular weights of carbohydrates on the structure and stability of spray-dried emulsions.

The influence of process parameters and feed composition on volatile retention during spray drying has been previously studied. As the diffusivity of the volatiles decreases rapidly with decreasing moisture content, most volatile loss occurs in the early stages of drying, before the formation of a dry crust at the surface of the drying droplets (Moreau and Rosenberg, 1993; Reineccius, 1988; Rosenberg and Sheu, 1996; Rulkens, 1973). Upon crust or skin formation, the outer layers of the drying droplet become low in moisture, which makes the skin virtually impermeable to the volatile compounds. To minimize volatile loss, a short constant-rate drying period before skin formation is thus preferred. However, subsequent drying leads to particle morphology changes, such as cracks and cavities, which may facilitate further volatile release (Bhandari et al., 1992; Siemons et al., 2020). For spray drying of emulsions, the formation of surface oil also affects the retention of volatiles in the spray-dried powder. It is thus expected that both (i) skin formation and (ii) particle morphology leading to surface oil formation, are expected to affect the retention of volatiles during encapsulation by spray drying.

The objective of this study is to understand how the DE value of maltodextrin influences volatile retention during encapsulation of emulsions by spray drying for which we address specifically the skin formation and morphology of particles. To the best of our knowledge, no research has previously studied this relationship and related phenomena, which are essential for optimizing the encapsulation of essential oils. We hypothesized that significant volatile losses occur before skin formation and that the DE value of maltodextrin determines the time until the skin is formed (i.e. locking point). Lower DE values, which are associated with higher viscosities, may facilitate earlier skin formation and thus reduce volatile loss. Conversely, higher DE values lead to lower viscosities and may delay skin formation, leading to increased volatile loss. To a certain extent, the morphological structure of particles may further promote the release of more volatiles during the rest of the drying period. This hypothesis is supported by the earlier findings related to the skin formation for different DE values conducted by Siemons et al. (2020).

To test this hypothesis, oil-in-water emulsions with a volatile in the dispersed phase were prepared. D-limonene was chosen as the model volatile due to its hydrophobic characteristic, which was then diluted in sunflower oil. This approach was used to mimic the typical concentration of d-limonene in essential oils (Miyazato, 2018). Pea protein isolate (PPI) was chosen as the emulsifier because of its well-known emulsifying properties and the shift towards more plant-based proteins to replace animal-based proteins (Hinderink et al., 2021). Maltodextrins of different DE values, namely DE 6, 12, 21, and 38, were used as carrier matrix to formulate the emulsions, which were then subjected to spray drying. The spray-dried emulsions were characterized, including their morphological properties and d-limonene retention. The results from this study contribute to a better understanding of the mechanism underlying volatile release during spray drying, specifically establishing to what extent skin formation and morphological development of particles determine overall volatile retention at the end of the spray drying process. This will help to better understand and steer the encapsulation of volatiles in the food industry.

## 2 Materials and methods

### 2.1 Materials

Pea Protein Isolate (PPI) and maltodextrins with Dextrose Equivalent (DE) 6, 12, 21, and 38 were purchased from Roquette Fréres (Lestrem, France). Sunflower oil was sourced from a local supermarket (Jumbo). Milli-Q water (Q-pod, Merck Millipore, Darmstadt, Germany) was used to prepare all emulsions. D-limonene with 97 % purity served as the model volatile, and analytical grade methanol, acetonitrile, isopropanol, n-hexane, and sodium dodecyl sulfate (SDS) were obtained from Sigma Aldrich (St. Louis, Missouri, USA).

### 2.2 Encapsulation process

#### 2.2.1 Stripping sunflower oil

Sunflower oil was stripped of undesired volatiles by passing nitrogen gas through 500 ml of oil in a 1-L Schott flask for 24 h at room temperature. After stripping, 100 ml of the oil was stored in a sealed plastic container at −20 °C until needed for experiments. The oil was thawed overnight at 4 °C before use.

#### 2.2.2 Emulsification

Four emulsions were prepared using different DE values of maltodextrin. Based on a wet basis, the initial composition was 20 wt% of solids, 0.9 wt% of protein content, and 6 wt% of oil content. A solids content of 20 wt% was selected to extend the constant-rate drying period, thereby facilitating the influence of DE on skin formation, particle morphology, and overall particle formation to be more clearly observed. Both the emulsion formulation and the drying conditions were kept constant throughout the experiment. A total of 700 ml of emulsion was made for each emulsification batch.

To prepare the formulations, PPI and maltodextrin were blended with demineralized water to the desired solids content using a high-speed blender (S18N-19G, Ultra-turrax R, IKA-Werke GmbH & Co., Staufen, Germany) at 15000 rpm for 5 min. The mixtures were refrigerated at 4 °C for 10–12 h to ensure full hydration. On the same day as emulsification and spray drying, the oil phase was prepared by dissolving d-limonene in stripped sunflower oil to a concentration of 7500 ppm by stirring using a magnetic stirrer (IKA RCT Basic, Germany) at 500 rpm for 20 min at room temperature in a closed glass bottle (Schott Duran 100 ml, Germany). After hydration, PPI and maltodextrin solution were mixed with the oil phase using a high-speed blender at 1500 rpm for 5 min to form a coarse emulsion, with the mixture kept inside an ice bath to minimize d-limonene loss. The emulsion was further homogenized using a microfluidizer (M-110Y Microfluidizer equipped with an F12Y interaction chamber, Microfluidics, Massachusetts, USA) at 1200 bar, repeated four times while cooling the outlet stream with ice water. The final emulsion was collected in a sealed glass bottle (Schott Duran 1000 ml, Germany) and stored at 4 °C for 2–4 h before spray drying.

#### 2.2.3 Spray drying

The drying of all emulsions was performed using a Model B-290 mini spray dryer (BUCHI Labortechnik AG, Switzerland) equipped with a two-fluid nozzle for atomization. All emulsions were dried under the same operating conditions. The drying air flow rate was set at 6 × 10^5^ ml/min (corresponding to an aspirator rate of 95 %) and an atomization airflow rate of 10^4^ ml/min. The inlet temperature was 178–182 °C, and the outlet temperature was 90–95 °C, which was controlled by adjusting the liquid feed flow rate, depending on the viscosity of the liquid feed emulsion before spray drying. In this study, the liquid feed flow rate ranged from 5.7 to 9.6 ml/min, corresponding to the highest to the lowest DE values. The resulting spray-dried emulsions were collected and stored in sealed plastic containers at −20 °C before further analysis. Two independent spray-drying experiments were conducted for each sample variation.

#### 2.2.4 Redispersion

The spray-dried emulsions were dispersed in a 1 wt% SDS solution (resulting in 10 wt% solids, wet basis) to directly stabilize the released emulsion droplets and enable the determination of oil size inside the powder (Jauhari et al., 2025). Powders of 0.4 ± 0.01 g were mixed with 3.6 ml of 1 wt% SDS solution to achieve 10 wt% solids, then vortexed at 2500 rpm for 2 min to ensure complete redispersion but no droplet breakup. Redispersion was conducted for spray-dried emulsions with and without surface oil.

### 2.3 Molecular weight distribution analysis for maltodextrin

Molecular weight distributions of the different maltodextrins were determined by High Performance Liquid Chromatography (HPLC) using a Shodex KS-803 8.0 × 300 (mm) IDxLength + Guard column. The column is operated at 50 °C and connected to a refractive index (RI) detector (Shodex RI-501). Milli-Q water was used as the eluent with a flow rate of 1 ml/min.

### 2.4 Emulsion analysis

#### 2.4.1 Droplet size

Oil droplet size distribution was measured using static light scattering, Mastersizer 3000 (Malvern Instruments Ltd., Worcestershire, UK) equipped with a wet dispersion unit. The Mie Theory was applied as the scattering model for droplet size analysis. Sunflower oil has a refractive index of 1.465, an absorption index of 0.01, and a density of 0.92 × 10^3^ kg/m^3^. Water with a refractive index of 1.330 is used as the dispersant in the cell chamber for measurement. For emulsions showing a bimodal distribution, aliquots were diluted 1:1 (v/v) in 1 wt% SDS solution to disrupt flocs before the measurement. Emulsions were analyzed within 2 h after emulsification and after 1 day to assess stability. The droplet size of redispersed spray-dried emulsions was measured immediately after redispersion, with additional measurements comparing spray-dried emulsions with and without surface oil. Size distributions were reported as volume distributions. Droplet size was the average of measurements from at least two independent samples, each measured five times.

To assess the flocculation instability of the initial emulsion after 1-day storage, the flocculation percentage (*%F*) was calculated based on the ratio of d [3,2] of droplet emulsion measured at day 0 (*E0*) and one day after storage at 4 °C (*E1*) without the addition of SDS solution, following Equation (1).

(1) $$\%F=\frac{E1-E0}{E0}\;\cdot 100\%$$

#### 2.4.2 Viscosity

The viscosity of the emulsions was measured using a strain-controlled rheometer (MCR 520, Anton Paar, Austria) with a double-gap concentric cylinder geometry (C-DG26.7/T200, Anton Paar, Austria) at 20 °C. The shear rate was logarithmically increased from 0.1 to 1000 s^−1^ across 20 measuring points, with durations decreasing logarithmically from 300 to 1 s. The process was then reversed, reducing the shear rate from 1000 to 0.1 s^−1^ with increasing durations of 1–300 s. From both measurements, a representative apparent viscosity was calculated at a shear rate of 144 s^−1^.

### 2.5 Analysis of spray-dried emulsions

#### 2.5.1 Yield and moisture content

The yield (%) was determined by dividing the mass of the powder collected after spray drying (*m*_*p*_) by the total solid mass of the initial emulsion before spray drying (*m*_*i*_), as presented in Equation (2).

(2) $$Yield=\frac{m_{p}}{m_{i}}\;\cdot 100\%$$

Moisture content was measured via oven drying. Approximately 1 g of spray-dried powder was placed in a hot air oven (Heratherm Oven, Langenselbold, Germany) at 105 °C for 12–18 h. The sample weight was recorded before ($m_{p}$) and after drying ($m_{f}$). The moisture content (%) was calculated on a dry basis using Equation (3).

(3) $$Moisture\;Content=\frac{m_{p}-m_{f}}{m_{f}}\;\cdot 100\%$$

#### 2.5.2 Particle size and specific surface area

Particle size distribution and specific surface area of the spray-dried emulsions were measured using a Mastersizer 3000 (Malvern Instruments Ltd., UK) with a dry dispersion unit. Scattering analysis was based on Mie Theory, using the refractive index of maltodextrin (1.673), the main powder constituent. To prevent agglomeration, a dispersion pressure of 2.0 bar and a high-energy Venturi were applied, with laser obscuration maintained between 1 and 10 %. Particle size was reported as volume mean diameter (d [4,3]) and measured in triplicate for each sample. Specific surface area (*SSA*) was calculated using the d [3,2] value and the skeletal density of each spray-dried emulsion.

#### 2.5.3 Surface oil

The amount of surface oil was determined via UV–vis spectrophotometry, adapted from the method initially proposed by Salimi et al. (2018) with some modifications. Two grams of spray-dried emulsion were dissolved in 12 ml of n-hexane in a 50 ml plastic centrifugation tube and vortexed (Digital Vortex Mixer, Fisher Scientific, USA) at 2500 rpm at room temperature for 1 min to extract non-encapsulated oil from the surface of the spray-dried emulsions. The solution was then centrifuged (Thermo Fisher Scientific, Legend XFR centrifuge, USA) at 4700 xG for 10 min at 20 °C. The supernatant was filtered through a 5.00 μm, 22 mm syringe filter (Milipore, Cork, Ireland), and 2 ml of the clear filtrate was transferred to a measuring cuvette (1 mm square cuvette) and analyzed using a UV–vis spectrophotometer (DR6000, Hach, Germany). The oil content was determined at a wavelength of 268 nm, where the sunflower in n-hexane shows the highest peak absorbance. The measured absorbance was correlated to sunflower oil content using a calibration curve made with known oil concentrations in hexane. Each sample was analyzed in triplicate, with five repetitions per measurement. The surface oil percentage (SO) was expressed as the mass percentage of extracted surface oil relative to the total oil added to the emulsion, with encapsulated oil calculated as the difference between total oil and surface oil. According to Linke et al. (2021), the oil load (g per g dry solids) at the end of the spray drying process is similar to the amount initially added to the emulsion. Therefore, in this study, we assumed that the oil load remained constant during the spray drying process. Consequently, the total oil used to calculate the surface oil percentage was based on the initial total oil amount added to the emulsion. The mass of surface oil per unit surface area of spray-dried emulsions ($m_{SO}\;per\;SA$, g/m^2^) was calculated based on the value of the specific surface area (SSA, m^2^/g) and mass of surface oil per powder ($m_{SO}\;per\;m_{powder}$ g/g), following Equation (4).

(4) $$m_{SO}\;per\;SA=\frac{m_{SO}\;per\;m_{powder}}{SSA}$$

#### 2.5.4 Skeletal density

The skeletal density of the spray-dried emulsions was measured using a pycnometer (Ultrapyc 1200e, Quantachrome) at a constant temperature of 20 ± 0.1 °C, with nitrogen as the displacement gas. Approximately 1 ± 0.2 g of the powder was placed in a metal cup for density analysis. Five data points (mass, volume, and density) were collected for each measurement, and the average value was calculated.

#### 2.5.5 Retention of d-limonene

The quantification of d-limonene was conducted using HPLC (Thermo Ultimate 3000 HPLC, ThermoFisher Scientific, Waltham, USA) with a Gemini 3 μm C18 (150 × 4.6 mm) column (Phenomenex, Torrance, USA). The column operated at 30 °C and was connected to a UV–Vis DAD detector, which detected d-limonene at 210 nm. The mobile phase consisted of methanol and acetonitrile in a 1:1 (v/v) ratio at a flow rate of 1.5 ml/min for 50 min. A calibration curve was constructed by dissolving d-limonene in n-hexane at concentrations from 0.005 to 0.25 mg/ml.

The extraction of d-limonene from the spray-dried emulsions was performed according to the procedure described by Jauhari et al. (2025). First, 0.4 g of powder was diluted in 1.6 ml of Milli-Q water in a 15-ml plastic centrifugation tube to achieve a solid content of 20 wt%. The mixture was then gently shaken by hand to ensure even dispersion, followed by vortexing at 2500 rpm for 1 min and rotation (Stuart, UK) at a speed of 40 rpm for 20 min at room temperature. Subsequently, the extraction was conducted by adding 2 ml of the mixture and 8 ml of solvent to a 15-ml centrifuge tube. The liquid was vortexed three times for 20 s each (with a 20-s rest in between). The mixture was then centrifuged at a speed of 4700 xG under ambient conditions for 40 min. The volume of the clear n-hexane phase (upper phase) was measured, and 1 ml was injected into an amber glass vial for further analysis using HPLC.

Several assumptions were made for calculations: (1) only d-limonene was assumed to be lost during encapsulation; (2) extracted d-limonene was assumed to be in sunflower oil, not the matrix; (3) sunflower oil was considered non-volatile and thus retained; and (4) mass balance calculations for d-limonene were based on HPLC results and the above-mentioned assumptions.

The d-limonene retention after spray drying ($R_{SD}$) was defined as the mass percentage of extracted d-limonene after spray drying ($Li_{SD}$) relative to the d-limonene extracted from the emulsion after emulsification ($Li_{Em}$), as per Equation (5). During the emulsification part of the d-limonene is already lost (Jauhari et al., 2025). To calculate the exact loss of d-limonene during spray drying ($Li_{loss\_SD}$), one therefore has to know the d-limonene content after emulsification and determine the loss during spray drying relative to that value. The loss of d-limonene during spray drying was thus determined by subtracting the d-limonene retention after spray drying from the d-limonene percentage extracted from the emulsion (100 %), following Equation (6).

(5) $$R_{SD}=\frac{Li_{SD}}{Li_{Em}}\;\cdot 100\%$$

(6) $$Li_{loss\_SD}=100\%-R_{SD}$$

#### 2.5.6 Scanning electron microscopy (SEM)

Micrographs of spray-dried emulsions were prepared using a SEM (JEOL JCM-7000, JEOL, Germany). Spray-dried emulsions were fixated on aluminum sample stubs (9.5 mm) using double-sided carbon adhesive tabs. Before imaging, the samples were coated with gold using a sputter coater (Joel Smart-Coater). SEM images were taken at 5 kV and viewed under a magnification of 1000× and 2000x.

### 2.6 Statistical and data analysis

Each emulsion and spray-dried emulsion was produced in duplicate, and the analysis was performed in triplicate for each independent sample. Statistical analysis was conducted using OriginPro software (OriginLab Corp., Northampton, USA), employing one-way ANOVA and Tukey's post hoc test to identify significant differences (p < 0.05). The data is presented as mean ± standard deviation.

## 3 Results and discussion

First, we will discuss the properties of initial emulsions (viscosity, droplet size, and stability) with different DE values (DE6, 12, 21, and 38). Followed by the characterization of the resulting spray-dried emulsions and the particle morphology, to elucidate its correlation with surface oil formation. Afterward, the changes in oil droplet stability during drying are discussed. Finally, the retention of d-limonene in the final powder will be discussed and linked to the properties of spray-dried emulsions influenced by the DE value of maltodextrin.

### 3.1 Characterization of the initial emulsions

The viscosities of the initial emulsions were assessed between a shear rate of 10 and 1000 s^−1^ (Fig. S1). The apparent viscosity significantly increased as the maltodextrin DE value decreased. The formulation with DE6 exhibited the highest viscosity (6.7 × 10^−3^ Pa s), while the DE38 emulsion was the lowest (2.7 × 10^−3^ Pa s). Intermediate values were measured for emulsions containing DE12 (4.0 × 10^−3^ Pa s) and DE21 (3.2 × 10^−3^ Pa s). This viscosity difference can be explained by the increased intermolecular interactions for larger polymer chains corresponding to the lower DE value (Fig. S2 (Heiden-Hecht et al., 2021; Siemons et al., 2020).

**Fig. 1:**
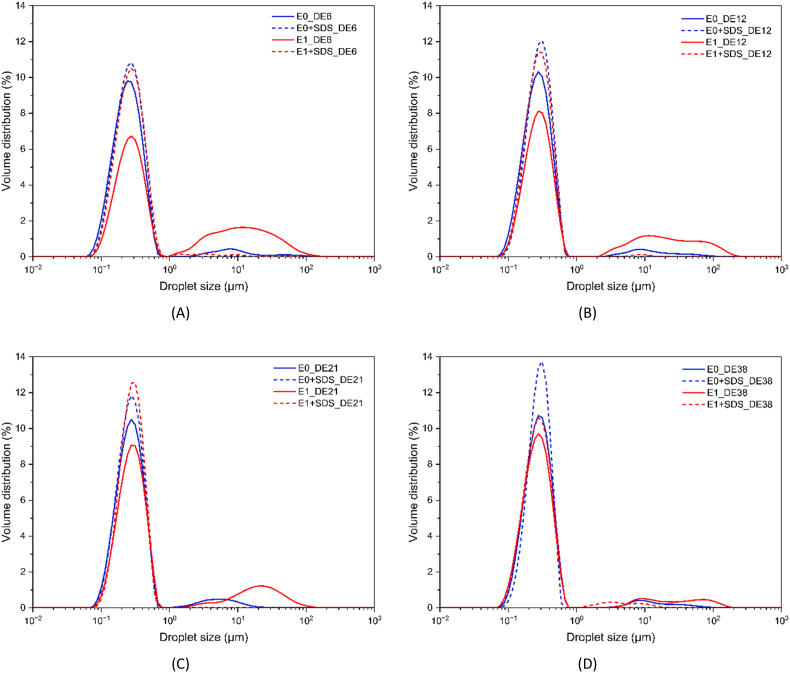
Volume-based size distributions of oil droplets in the initial emulsions containing maltodextrin of DE6 (A), DE12 (B), DE21 (C), and DE38 (D) at day 0 (E0, blue lines) and after one day of storage at 4 °C (E1, red lines). Dashed lines represent the oil droplet size distributions after 1:1 (v/v) dilution of 1 wt% SDS solution.

The average droplet size of the initial emulsions was similar regardless of the DE value (E0, Table 1). A smaller broad secondary peak above 1 μm was observed for droplet distributions of all emulsions (E0, Fig. 1), which disappeared upon the addition of 1 wt% SDS to the emulsion (E0+SDS), indicating some flocculation. Increasing viscosity due to a lower DE value did not appear to affect the droplet size of the initial emulsions.

**Table 1:** Sauter mean diameter (d [3,2]) of the initial emulsion measured immediately after emulsification at day 0 (E0), after 1:1 (v/v) dilution of 1 wt% SDS solution (E0+SDS); and after one day of storage at 4 °C without (E1), and with 1:1 (v/v) dilution of 1 wt% SDS solution (E1+SDS). The percentage of flocculation (%F) is calculated based on Equation (1).

Sample Name	*d[3,2]* (μm)				*%F*
	E0	E0+SDS	E1	E1+SDS	
**DE6**	0.24 ± 0.02 ^b,B^	0.24 ± 0.01 ^c,B^	0.41 ± 0.12 ^a,A^	0.26 ± 0.01 ^b,B^	70.75 ± 3.74 ^a^
**DE12**	0.27 ± 0.02 ^a,B^	0.28 ± 0.01 ^a,B^	0.38 ± 0.10 ^a,A^	0.27 ± 0.01 ^a,B^	41.46 ± 2.96 ^b^
**DE21**	0.27 ± 0.03 ^a,B^	0.26 ± 0.01 ^b,B^	0.35 ± 0.10 ^a,A^	0.27 ± 0.01 ^a,B^	29.83 ± 3.34 ^c^
**DE38**	0.28 ± 0.01 ^a,B^	0.28 ± 0.02 ^a,B^	0.28 ± 0.10 ^a,A^	0.27 ± 0.03 ^a,B^	1.08 ± 0.54 ^d^

Lowercase letters indicate significant differences within the same column, while the same letters do not differ significantly (p < 0.05). Uppercase letters indicate significant differences within the same row, while the same letters do not differ significantly (p < 0.05).

After one day of storage at 4 °C, the second peak became more prominent, especially at the lower DE value of maltodextrin (E1, Fig. 1). This instability was attributed to flocculation as confirmed by similar droplet sizes (E1+SDS) to the initial emulsion (E0+SDS) after SDS solution addition (ratio of emulsion to 1 wt% SDS solution is 1:1 (v/v)). The flocculation percentage (%F) was calculated by Equation (1) and was quantified for all prepared emulsions, as shown in Table 1. The value of %F significantly increased as the DE value decreased, indicating a reduced stability. The consistent findings were also confirmed by Kurtz et al. (2025) who reported that for lower protein content, emulsions prepared with low DE maltodextrin (DE6) exhibited higher viscosity but produced larger droplet sizes after homogenization compared to those with higher DE maltodextrin (DE40). This can be explained by the lower surface-active properties and higher viscosity of higher molecular weight maltodextrins, reducing protein mobility during emulsification, which altogether negatively contributes to emulsion stability (Heiden-Hecht et al., 2021).

### 3.2 Characterization of the spray-dried emulsions

Each emulsion was subjected to spray drying, and the resulting powders were analyzed. The particle size distributions (Fig. S3) and average particle sizes (Table 2 and Table S1) were the same across all samples, regardless of the DE value of maltodextrin used. This uniformity was attributed to the standardized initial solid content of 20 wt% across the formulations, despite a slight increase in the viscosities of the initial emulsion (Vicente et al., 2013). In two-fluid nozzles, the Sauter mean diameter is mainly influenced by the atomizing-gas flow (often expressed as the gas-to-liquid flow-rate ratio), while the effect of increasing liquid feed flow rate is comparatively weak – especially at higher atomizing gas flow, as in our study (Focaroli et al., 2019; Hede et al., 2008; Poozesh et al., 2018, 2020; Vidovič et al., 2022). The yield was consistent at around 60 % for all spray-dried emulsions due to similar losses in the spray-drying process. The moisture content for powders remained uniformly below 5 %, indicating that all were in the glassy state.

**Table 2:** General properties of the spray-dried emulsions.

Sample Name	DE6	DE12	DE21	DE38
*d[3,2] (μm)*	3.97 ± 1.01 ^A^	3.41 ± 0.14 ^A^	2.92 ± 0.07 ^A^	3.96 ± 1.15 ^A^
*d[4,3] (μm)*	6.97 ± 0.28 ^C^	8.53 ± 0.23 ^B^	6.36 ± 0.17 ^C^	10.04 ± 2.13 ^A^
*dx(50) (μm)*	6.17 ± 0.17 ^B^	6.35 ± 0.12 ^B^	5.70 ± 0.14 ^C^	6.63 ± 0.26 ^A^
*Yield (%)*	62.9 ± 0.82 ^A^	61.5 ± 0.08 ^A^	60.3 ± 0.86 ^A^	60.1 ± 2.22 ^A^
*Moisture Content (%)*	0.48 ± 0.28 ^A^	0.77 ± 0.34 ^A^	1.13 ± 0.67 ^A^	0.64 ± 0.15 ^A^
$\rho_{skeletal}$^a^*(g/cm*^*3*^*)*	1.19 ± 0.01 ^A^	1.20 ± 0.01 ^A^	1.21 ± 0.01 ^A^	1.21 ± 0.01 ^A^

Uppercase letters indicate significant differences within the same row, while the same letters do not differ significantly (p < 0.05).

a Before surface-oil stripping.

### 3.3 Change in oil droplet size distribution during spray drying

**Fig. 2:**
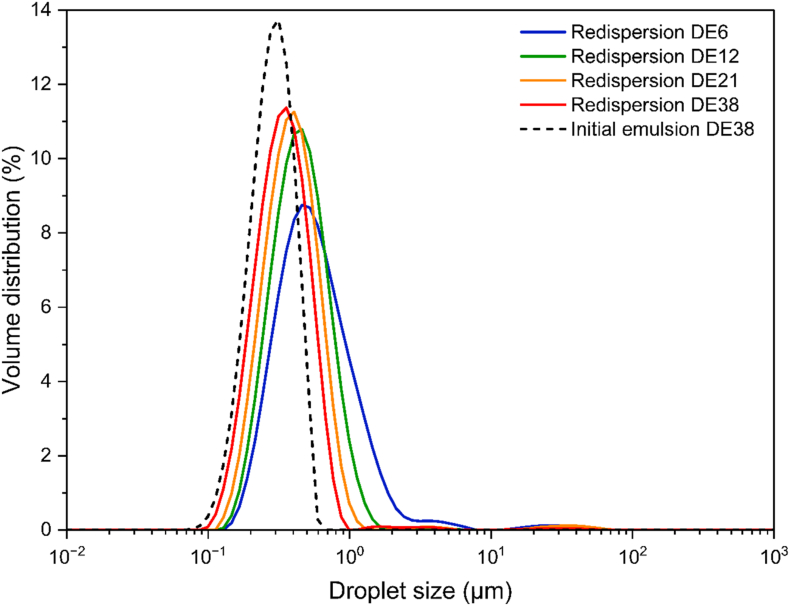
Volume-based size distributions of oil droplets in spray-dried emulsions containing surface oil, redispersed in 1 wt% SDS solution. Formulations are prepared with maltodextrin of DE6 (blue solid line), DE12 (green solid line), DE21 (yellow solid line), and DE38 (red solid line). The black dashed line represents the oil droplet size distribution of the initial emulsion containing DE38, diluted 1:1 (v/v) in 1 wt% SDS solution, and is shown as a representative reference for all initial emulsions.

**Fig. 3:**
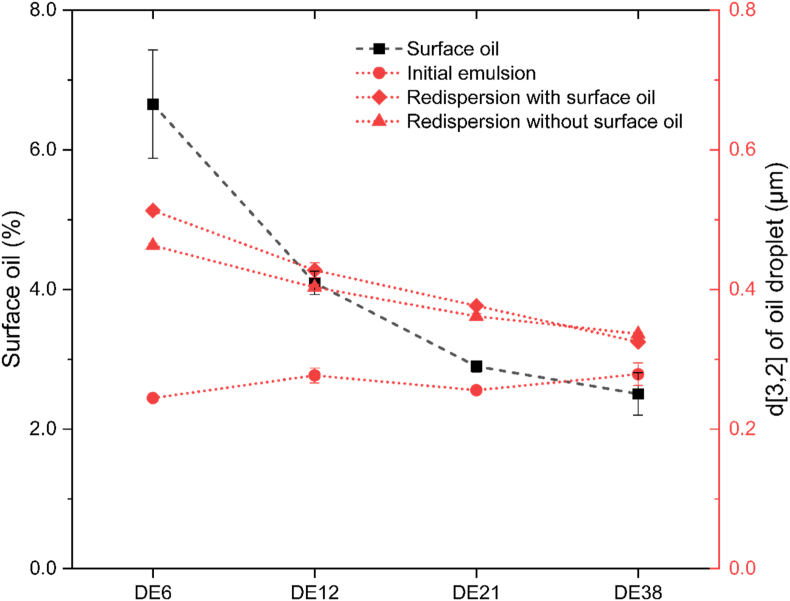
Correlation between surface oil percentage (black squares) and Sauter mean diameter of oil droplets in: initial emulsions diluted 1:1 (v/v) in 1 wt% SDS solution (red circles), spray-dried emulsions containing surface oil redispersed in 1 wt% SDS solution (red diamonds), and spray-dried emulsions without surface oil redispersed in 1 wt% SDS solution (red triangles), for formulations prepared with different DE values of maltodextrin. Error bars display the standard deviations. Lines were added as a visual guide.

The DE value of maltodextrin may affect the stability of the oil droplet size during the spray drying process. Fig. 2 shows the volume distribution of oil droplet size before spray drying and after reconstitution of the spray-dried emulsions, whereas Fig. 3 visualizes the correlation between the surface oil percentage and the average Sauter mean diameter of the oil droplets in the reconstituted powder for different DE values of maltodextrin. Initially, the oil droplets in all emulsions were comparable in size (E0, Table 1). However, after spray drying, an increase in droplet size was observed, particularly as the DE value of maltodextrin decreased (Fig. 2). A broader oil size distribution was also observed at lower DE values, as indicated by span values of ∼1.9, ∼1.3, ∼1.2, and ∼1.1 for DE6, DE12, DE21, and DE38, respectively (Table S2). The change in oil droplet size distribution may be related to the presence of more surface oil or the properties of the specific maltodextrin used as wall material.

To evaluate the influence of surface oil, we compared redispersed spray-dried emulsions with and without surface oil, as shown in Fig. 3, Fig. S6, and Table S2. Results showed that removing surface oil slightly reduced droplet size in samples with lower DE values (DE6 and DE12), although the reduction was not significant. Meanwhile, a significant increase in oil droplet size was observed when comparing the oil droplet size of initial emulsions to that of redispersed spray-dried emulsions without surface oil. This suggests that the coalescence of the encapsulated droplets is likely the primary cause of the increase in droplet size after spray drying, rather than surface oil formation on the particles.

The properties of maltodextrin (i.e., molecular weight and viscosity) are likely the primary factors affecting the observed changes in oil droplet size during the spray drying. Initial emulsions containing lower-DE maltodextrin already exhibited lower stability against flocculation before it is subjected to spray drying, as indicated by a higher value of %F (Table 1). The increased temperatures during spray drying also contribute to the destabilization of the emulsion and lead to the coalescence of oil droplets, particularly if the drying droplets are not yet fully solidified. As a result, larger coalesced encapsulated oil droplets were found inside the final spray-dried emulsions. A similar finding was also done in an earlier study, showing that less stable initial emulsions (due to lower emulsifier content) resulted in larger coalesced oil droplets inside final spray-dried emulsions (Jauhari et al., 2025).

Although the surface oil is not the primary factor influencing changes in oil droplet size during spray drying, a positive correlation between the oil droplet size inside the spray-dried emulsions and the surface oil percentage was observed (Fig. 3). As the use of lower DE maltodextrin resulted in larger oil droplets in the particles during drying, the probability that those oil droplets are in contact with the particle surface increases, leading to a higher surface oil percentage. This trend aligns with the findings by Linke et al. (2020), who observed that larger oil droplets inside the spray-dried particle, measured by NMR, contribute to more surface oil. Similarly, Ghani et al. (2017) reported a relationship between the encapsulation efficiency and the diameter ratio of water-based reconstituted emulsion droplets to spray-dried particles. Jafari et al. (2007) also confirmed that increasing the diameter ratio of the initial emulsion droplets to the powder particles reduces surface oil.

### 3.4 Surface oil formation: particle density and morphology

In preliminary experiments, the extraction time for surface oil of different DE values was determined after 1, 5, 10, and 15 min (Fig. S4). The SO of all spray-dried emulsions after n-hexane extraction was similar for all different extraction times. It was concluded that all surface oil is effectively extracted after a short extraction time of 1 min. These results align with Ghani et al. (2017), who also reported consistent surface oil levels after a short extraction time using n-hexane solvent. The SO of the spray-dried emulsions showed a noticeable decrease with an increase in the DE value of the maltodextrin used as the wall material (Table 3). A similar trend was also observed for the mass of surface oil per surface area (m_SO_ per SA) of the spray-dried particles. The highest m_SO_ per SA was found in the spray-dried emulsion with DE6 (∼1.7 %), while the lowest was for DE38 (∼0.6 %). Initially, it was hypothesized that lower DE values would lead to reduced surface oil content, explained by the earlier skin formation (e.g., locking point) during the spray drying process for lower DE values (Siemons et al., 2020). Earlier skin formation would prevent oil migration towards the surface for the rest of the drying period. However, the current results contradicted this hypothesis, showing that lower DE values were associated with increased surface oil content.

**Table 3:** Mass percentage of surface oil (SO), mass ratio of surface oil to powder ($m_{SO}\;per\;m_{powder}$), specific surface area (SSA), and mass of surface oil per unit surface area ($m_{SO}\;per\;SA$) of the spray-dried emulsions.

Sample Name	*SO* (%)	$m_{SO}\;per\;m_{powder}$. 10^3^ (g of surface oil per g of powder)	*SSA* (m^2^/g)	$m_{SO}\;per\;SA$. 10^3^ (g of surface oil per m^2^ of powder's surface area)
**DE6**	6.65 ± 0.77 ^a^	1.98 ± 0.23 ^a^	1.33 ± 0.30 ^b^	1.69 ± 0.76 ^a^
**DE12**	4.09 ± 0.17 ^b^	1.22 ± 0.05 ^b^	1.47 ± 0.07^ab^	0.83 ± 0.02 ^b^
**DE21**	2.90 ± 0.08 ^c^	0.86 ± 0.02 ^c^	1.70 ± 0.04 ^a^	0.51 ± 0.01 ^c^
**DE38**	2.50 ± 0.30 ^d^	0.75 ± 0.09 ^d^	1.38 ± 0.30 ^b^	0.56 ± 0.08 ^c^

Different letters indicate significant differences within the same column (p < 0.05).

**Fig. 4:**
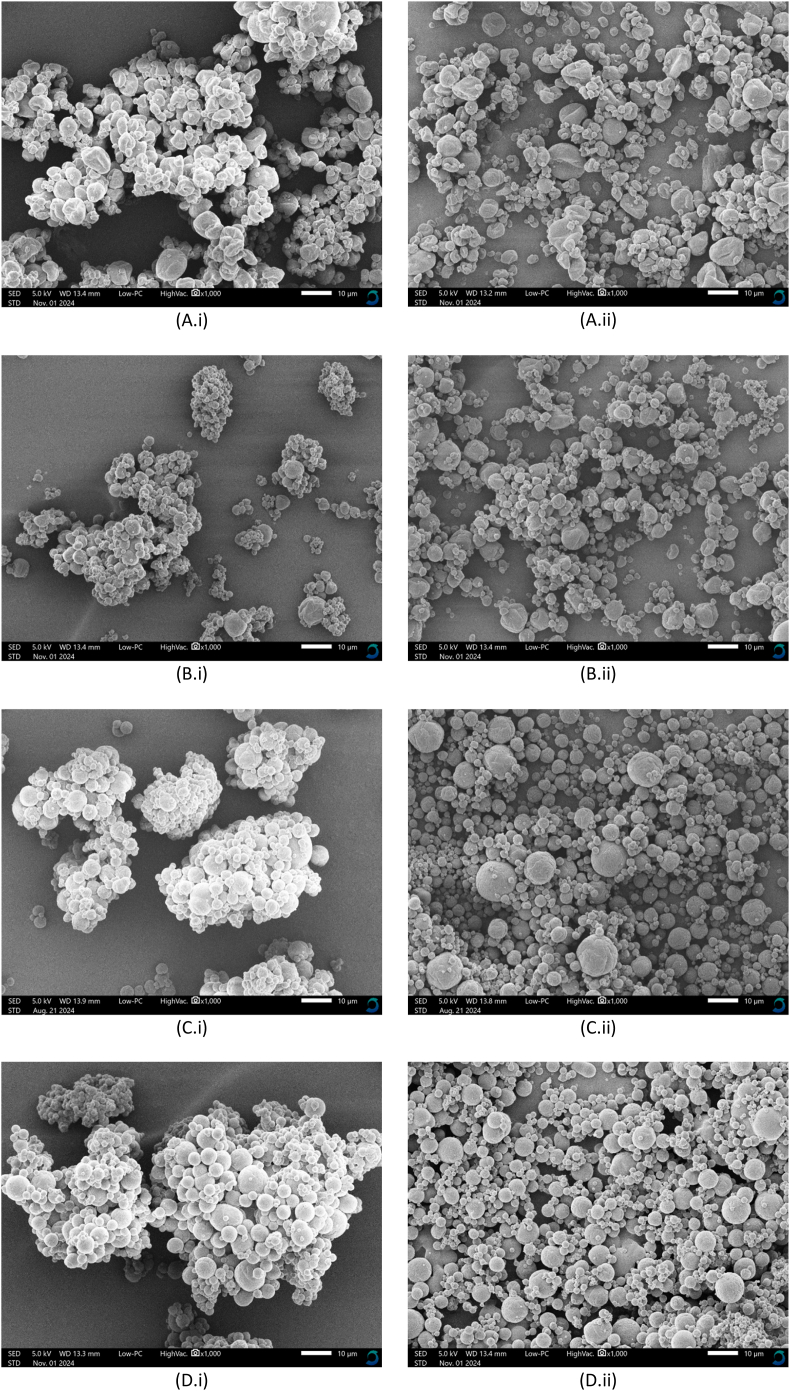
Morphology of the spray-dried emulsions containing maltodextrin of DE 6 (A), DE12 (B), DE21 (C), and DE38 (D), shown with (i) and without (ii) the surface oil. All figures have the same magnification of 1000×, with the scale bars indicating 10 μm.

To understand the influence of the DE value of maltodextrin on the mechanism of surface oil formation, subsequently, the morphology of particles before and after surface oil stripping (Fig. 4) were analyzed. The skeletal density measured for all powders before stripping was similar, ∼1.2 g/cm^3^ (Table 2), and did not change significantly after stripping, especially for DE21 and DE38 (data not shown). However, the surface oil of the particles with lower DE values (DE6 and DE12) was higher than that of the higher DE values (DE21 and DE38) (Table 3). This suggests that particles with lower DE maltodextrins have a more open porous surface structure than those with higher DE values, allowing surface oil to fill the surface pores. This observation aligns with the recent findings by Kurtz et al. (2025), who quantified surface free fat content using a different method – n-heptane extraction followed by gravimetric analysis. The authors reported that for the fine emulsion droplets, a similar decreasing trend in surface oil content was observed as the DE value of maltodextrin increased from 6 to 40. Earlier studies confirm that a reduced particle density is correlated with earlier crust formation (Bhandari et al., 1992), in which decreasing the DE value of maltodextrin promotes an earlier locking point for pure maltodextrin particles (Siemons et al., 2020).

SEM images further revealed that significant particle agglomeration occurred in all samples where surface oil was present (Fig. 4i). The extent of agglomeration was reduced after the removal of the surface oil (Fig. 4ii). Removing the surface oil also allowed for better observation of the morphological structure of particles with different DE values. Powders with lower DE values (DE6 and DE12) had a smoother surface but were less spherical due to the presence of larger and deeper dents. In contrast, powders with higher DE values (DE21 and DE38) demonstrated more spherical shape but rough surface with smaller and shallower dents.

In this study, no obvious holes could be observed on the particle surfaces in the SEM images (Fig. 4), even at a magnification of 2000× (Fig. S5). This finding aligns with previous work by Li et al. (2020) and Peng et al. (2023), who similarly reported the absence of visible holes or cracks in SEM images. However, Jafari et al. (2007) occasionally identified cracks on the surfaces of particles prepared using Hi-Cap 100 and whey protein concentrate, which are attributed to weak viscoelastic properties of the wall material at the final stages of drying. Large particles frequently had large dents or concavities and mostly presented with cracks. Supporting this, Linke et al. (2020) also observed dark spots on the surface of spray-dried particles after surface oil removal, which may indicate the presence of holes previously filled with oil.

**Fig. 5:**
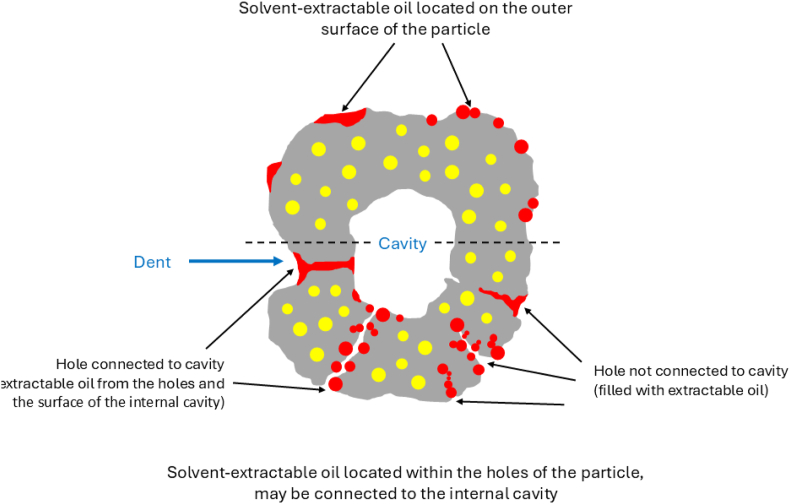
Schematic model representation of the cross-sectional area of a spray-dried particle illustrating different locations of solvent-extractable surface oil. The upper section shows the solvent-extractable oil located on the outer surface of the particle, while the lower section shows the solvent-extractable oil located within the holes of the particle, which may or may not be connected to the internal cavity. Red and yellow colors indicate solvent-extractable surface oil and encapsulated oil, respectively. Adapted from Vega and Roos (2006) and Vignolles et al. (2007).

**Fig. 6:**
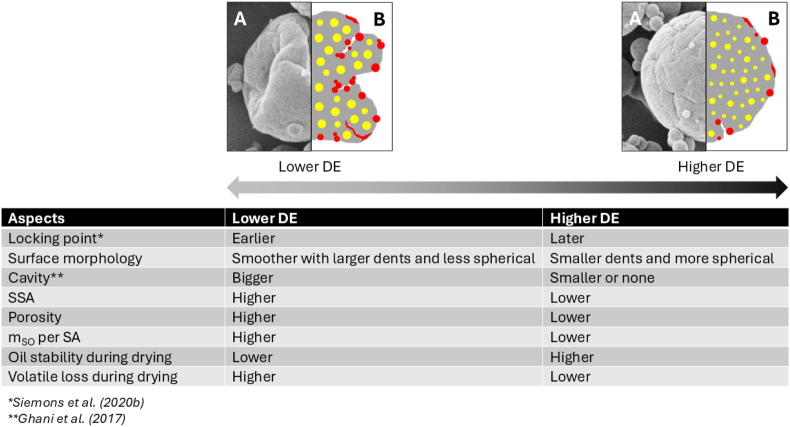
Overall properties of spray-dried particles containing d-limonene volatile, illustrated with (A) a representative SEM image, and (B) a schematic model of the cross-sectional area of the spray-dried particle, as influenced by the DE value of maltodextrin. Red and yellow colors on the schematic model indicate the solvent-extractable surface oil and encapsulated oil, respectively.

With the above-mentioned results, we show that the DE value of maltodextrin significantly influences the amount and the distribution of surface oil by affecting the morphology of particles during spray drying. The increased SO observed in samples with lower DE values is likely attributed to the structural changes of the drying droplet after the locking point. Wall materials with lower DE values generally exhibit rapid skin formation with more elastic properties that allow water evaporation from within the droplet (Siemons et al., 2020). Although the exact mechanism of cavity formation is unknown, it has been hypothesized that the elastic skin is compressed during further water evaporation, increasing the elastic energy of deformation. Elastic stress can then be released by the formation of holes, followed by cavity formation, consequently enlarging the particle surface area and enhancing the water evaporation process (Bouman et al., 2016). This results in collapsed structures, uneven shrinkage, and exposes the interior of the droplet. This process will likely continue until the entire droplet is solidified. As a result, formulations containing lower DE values exhibited bigger internal cavities, larger dents, and more holes, which contribute to a larger surface area of the spray-dried emulsions (Ghani et al., 2017; Takashige et al., 2020). This increasing number of cavities and holes leads to a reduced density of dried particles (De Vilder et al., 1976). It consequently increases the particle porosity, which is also supported by an earlier study on pure maltodextrin powders that show the increased porosity as the DE value decreased (Siemons et al., 2020). Therefore, surface oil could also have come from the interior of the particles through these holes besides the outer surface of spray-dried emulsions (Fig. 5), as a solvent extraction method is being used (Vega and Roos, 2006; Vignolles et al., 2007). This surface oil consequently contributes to a higher surface oil percentage for spray-dried emulsions with lower-DE maltodextrin (Fig. 6). In contrast, maltodextrin with higher DE values exhibits more viscous rather than elastic behavior, resulting in more gradual and uniform shrinkage of emulsified droplets during drying. The spray-dried particles have smaller internal cavities and are less porous (Ghani et al., 2017; Takashige et al., 2020). As a result, surface oil is mostly located only on the outer surface of the particle, contributing to a lower surface oil percentage of these powders (Fig. 6).

### 3.5 D-limonene loss during spray drying

**Fig. 7:**
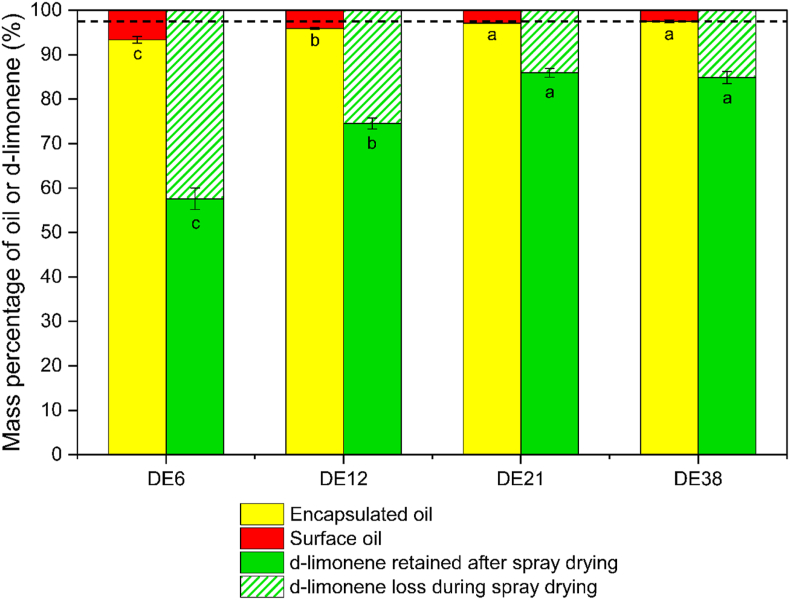
Mass percentages of oil and d-limonene after spray drying, compared to their initial amounts before spray drying, for formulations prepared with different DE values of maltodextrin. The black dashed line indicates the encapsulated oil percentage of the DE38 formulation and serves as a visual reference for comparison with other formulations. Error bars display the standard deviations, and the same letters for the same color bars do not differ significantly (p < 0.05).

The retention of d-limonene after spray drying was determined by calculating the mass percentage of d-limonene extracted from the spray-dried emulsion compared to the d-limonene extracted from the initial emulsion before drying, in which the mass percentage for all samples was still higher than 92 % (Table S3). The retention of d-limonene in spray-dried emulsions was reduced as the DE values of maltodextrin decreased (Fig. 7 and Table S4). The lowest retention was observed in the powders prepared with maltodextrin DE6, which retained only ∼57 % of d-limonene, while the highest retention (∼85 %) was achieved with maltodextrin DE21 and DE38. For all formulations, the percentage of d-limonene retention was consistently lower than that of encapsulated oil.

The loss of d-limonene from the surface oil was likely, as it was directly exposed to the environment during spray drying, resulting in rapid evaporation. In previous research, no d-limonene was detected in the surface oil when analyzed by HPLC (Jauhari et al., 2025). However, the current study indicates that the primary source of d-limonene loss was the encapsulated oil, with losses ranging from ∼12.6 % to ∼35.8 %, far exceeding those from surface oil (∼2.5–6.7 %) (Table S4). The d-limonene loss from encapsulated oil increases as the DE value of maltodextrin decreases.

With an initial solids content of 20 wt%, emulsion droplets experienced an extended constant-rate drying period. During this drying period, d-limonene (not only from surface oil but also from all emulsion droplets) can freely diffuse and evaporate into the environment. Once skin formation begins (e.g., at the locking point), the loss of d-limonene from the encapsulated oil significantly decreases, but release from the surface oil likely continues. The higher viscosity of lower-DE maltodextrin results in an earlier locking point and decreased relative diffusion of volatile (Menting et al., 1970; Siccama et al., 2023; Siemons et al., 2020). Consequently, if no additional factors can influence the further d-limonene release, it is expected that a higher d-limonene would be retained for the lower DE value of maltodextrin. However, the current findings contradict this hypothesis, indicating that other factors might influence the release of d-limonene after the skin is formed.

**Fig. 8:**
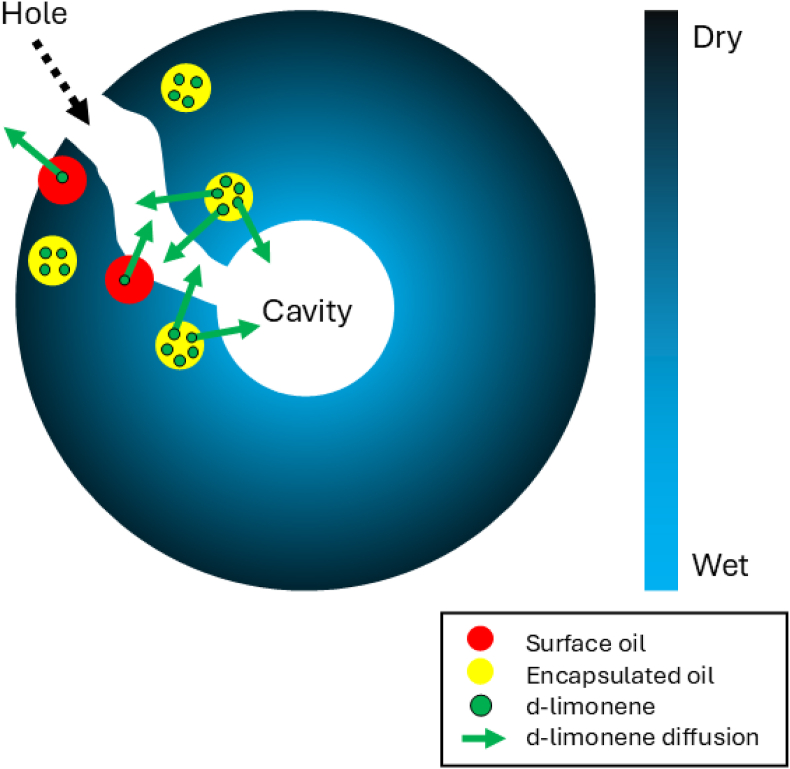
Schematic illustration of the volatile release mechanism from a drying emulsion droplet after skin formation due to the early formation of a hole and cavity during the spray drying. The color gradient from light blue to black indicates the moisture gradient within the droplet, transitioning from wet to dry matrix. Green circles represent d-limonene molecules, green arrows indicate the diffusion pathway of those from the oil phase to the surrounding air. Red and yellow circles represent the surface oil and encapsulated oil, respectively. The illustration is not drawn to scale.

Particle morphology can explain the more pronounced release of d-limonene from encapsulated oils observed in this study. The spray-dried emulsions containing DE6 resulted in the most porous particles, exhibited the highest surface oil percentage (∼6.7 %), and the highest d-limonene loss during drying (∼42.4 %), of which ∼35.8 % originated from the encapsulated oil fraction (Table S4). This shows that the timing of hole and cavity formation is particularly critical. Early formation of holes and large internal cavities, before the drying droplet fully solidifies, exposes additional surface oil. This exposure prolongs the constant-rate period (Bouman et al., 2016) and we hypothesize that it creates extra diffusion pathways, which provide more time and surface area for d-limonene release. Thus, d-limonene can be released from both surface and encapsulated oils until solidification is complete (Fig. 8). Consequently, the higher observed loss of d-limonene in spray-dried emulsions with lower DE values may be attributed to the formation of holes as well as larger and more cavities. Similar behavior, though less pronounced, is seen for DE 12, consistent with its lower d-limonene retention and porosity.

In contrast, particles containing higher-DE maltodextrin (DE21 and DE38) had similar surface oil percentage and d-limonene retention, which are higher than those from lower DE (DE6 and DE12). The d-limonene loss from encapsulated oil is similar for both particles, but is much higher than that from surface oil. Those particles are less porous with the distribution of the surface oil mostly on the outer surface of the particles. Thus, it is likely that the loss of d-limonene for a higher DE value is mostly related to the duration of the constant-rate drying period affected by the lower initial solid content of the emulsion (20 wt%). This reasoning is supported by an earlier study where an initial solid content of 40 wt% significantly reduced d-limonene loss from encapsulated oils (Jauhari et al., 2025).

Overall, these results demonstrate that the DE value of maltodextrin determines the d-limonene retention during spray drying primarily by influencing the particle morphology rather than the earlier locking point alone. Future studies should be directed towards a better understanding of the fundamental mechanisms governing d-limonene release during both the constant- and falling-rate drying periods. Emphasis could be put on how particle morphology development influences volatile retention, which may be studied via thin film drying experiments (Siccama et al., 2023). Confocal laser scanning microscopy (CLSM) analysis using a fluorescent volatile may be helpful to confirm the distribution of volatiles within both the encapsulated oil and surface oil of the dried emulsified particle, similar to the method used for identifying oxidation products (Yang et al., 2024). Furthermore, single droplet drying experiments can be performed, enabling the observation of morphology development of emulsified droplets, especially the formation of holes and cavities (Bouman et al., 2016), as well as the distribution of surface oil and volatiles during drying (Abd Ghani et al., 2017; Both et al., 2018).

## 4 Conclusion

To effectively encapsulate volatiles by spray drying, the choice of wall material is important as it determines the encapsulation efficiency and the quality of the final powders. In this study, we investigated volatile retention as influenced by the DE value of maltodextrin during spray drying. Four emulsions containing d-limonene loaded in sunflower oil were encapsulated with four different DE values of maltodextrin through the spray drying process. The properties of the initial emulsions and the spray-dried emulsions were assessed to understand the influence of maltodextrin's properties on the retention of d-limonene after the spray-drying process.

This study revealed that the d-limonene retention on different DE values of maltodextrin was not limited by skin formation; instead, the particle morphology that was correlated with the formation of surface oil played a crucial role in determining overall d-limonene retention at the end of the spray drying. When emulsions containing 20 wt% solids were dried using lower-DE maltodextrin (i.e., DE 6 and DE 12), the resulting powders exhibited higher surface oil percentage and reduced d-limonene retention, compared to higher-DE maltodextrin (i.e., DE 21 and DE 38). These results were attributed mainly to the formation of holes and cavities as the emulsion droplet skin formed and dehydrated. This observation was validated by reduced particle density after surface oil removal, indicating a more porous structure in samples with lower-DE maltodextrin. Cavity formation is suggested to create new pathways and additional surface areas that allow for substantial d-limonene losses, thereby challenging the initial hypothesis that lower-DE maltodextrin would promote d-limonene retention through rapid skin formation. The current findings emphasize that controlling the morphology and structural integrity of spray-dried emulsions during the spray drying is crucial for improving the retention of hydrophobic volatile compounds like d-limonene.

## Author contributions

Ana K.P. Jauhari: Conceptualization, Methodology, Writing – original draft, Data curation, Formal analysis, Visualization.

Viktorija Lucenko: Data curation, Formal analysis.

Meinou N. Corstens: Conceptualization, Methodology, Writing – review & editing, Supervision.

Patrick F.C. Wilms: Conceptualization, Methodology, Writing – review & editing, Supervision.

Maarten A.I. Schutyser: Conceptualization, Methodology, Writing – review & editing, Supervision, Project administration, Funding acquisition.

## Declaration of generative AI and AI-assisted technologies in the writing process

During the preparation of this work, the authors used Gemini to improve the readability of sentences in the manuscript. After using this tool, the authors reviewed and edited the content as needed and take full responsibility for the content of the publication.

## Declaration of competing interest

The authors declare that they have no known competing financial interests or personal relationships that could have appeared to influence the work reported in this paper.

## Glossary

Dent: A hollow located on the surface of the dried particle due to water evaporation during drying. It can be deep or shallow depending on the properties of the wall materials and drying conditions.
Cavity: A void or space inside the dried particle due to water evaporation during drying. Sometimes, it is also referred to as a vacuole.
Locking point: Transition time from constant-rate drying to falling-rate drying period, marked by the formation of dried skin on the particle surface.
DE: Dextrose equivalence
Tg: Glass transition temperature
PPI: Pea protein isolate
SDS: Sodium dodecyl sulfate
HPLC: High performance liquid chromatography
SSA: Specific surface area of powder
SO: Surface oil percentage
m_SO_ per SA: Mass of surface oil per surface area of the powder
m_SO_ per m_powder_: Mass ratio of surface oil to powder
E0: Emulsion day 0
E0+SDS: Emulsion day 0 diluted 1:1 (v/v) in 1 wt% SDS solution
E1: Emulsion day 1
E1+SDS: Emulsion day 1 diluted 1:1 (v/v) in 1 wt% SDS solution
