# Supplementary material for: Encapsulating volatiles by spray drying: The choice of dextrose equivalent influences d-limonene retention via skin formation and particle morphology
Source: Curr Res Food Sci. 2025 Oct 10;11:101224. doi: 10.1016/j.crfs.2025.101224 (PMC12550165; doi:10.1016/j.crfs.2025.101224)
Supplement: Multimedia component 1 [file mmc1.pdf]

Supplementary Materials

Encapsulating volatiles by spray drying: The choice of dextrose equivalent influences d-limonene retention via skin formation and particle morphology

Authors: Ana K.P. Jauhari[a], Viktorija Lucenko[a], Meinou N. Corstens[a], Patrick F.C. Wilms[a], Maarten A.I. Schutyser[a,*]

Affiliation:

[a] Laboratory of Food Process Engineering, Wageningen University & Research, P.O. Box 17, 6700 AA, Wageningen, The Netherlands

* Corresponding author: maarten.schutyser@wur.nl

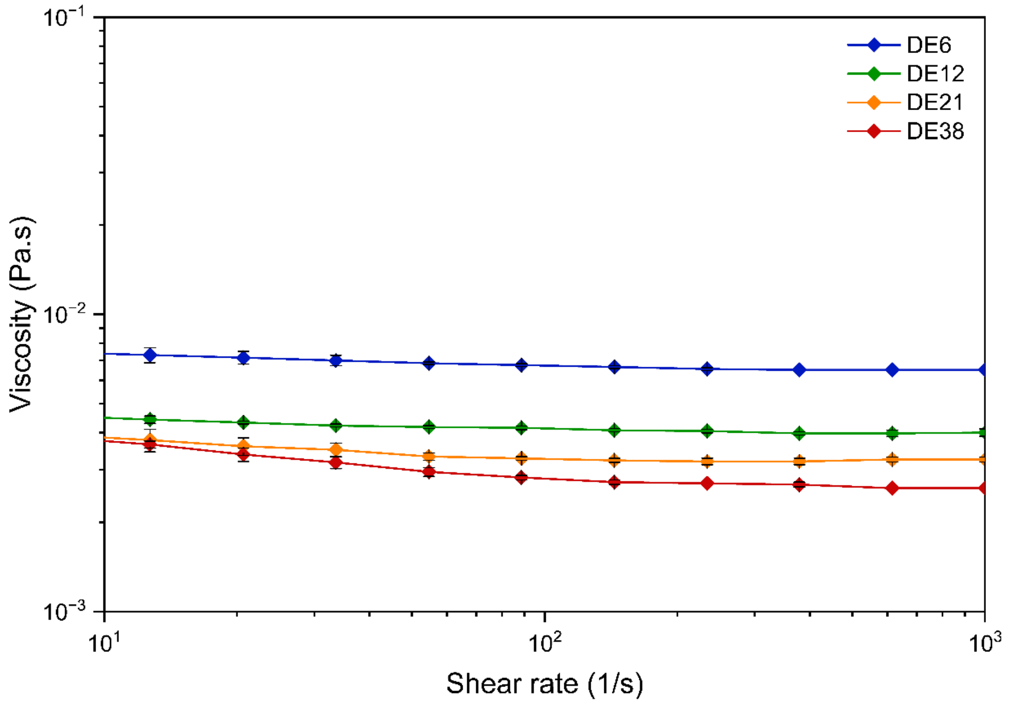

Figure S1. Viscosity of initial emulsions as a function of shear rate for formulation containing maltodextrin of DE6 (blue diamonds), DE12 (green diamonds), DE21 (yellow diamonds), and DE38 (red diamonds). Error bars display the standard deviations.

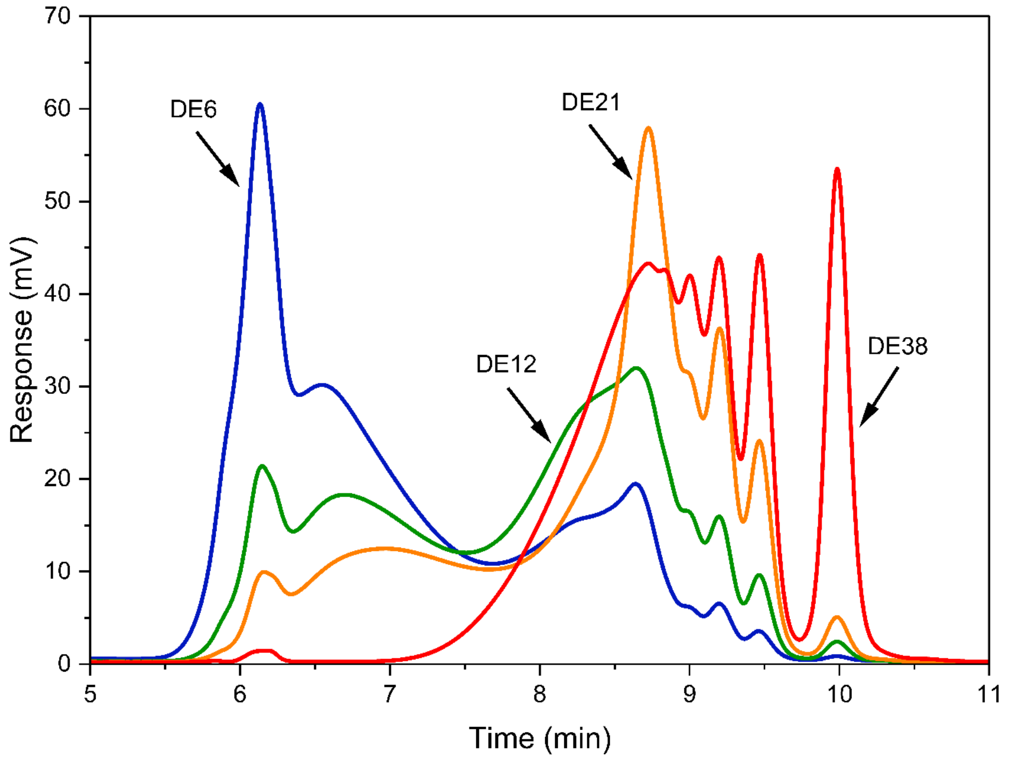

Figure S2. Molecular weight distribution for maltodextrin DE6 (blue line), DE12 (green line), DE21 (orange line), and DE38 (red line). Molecules with higher molecular weight elute first, followed by low molecular weight components.

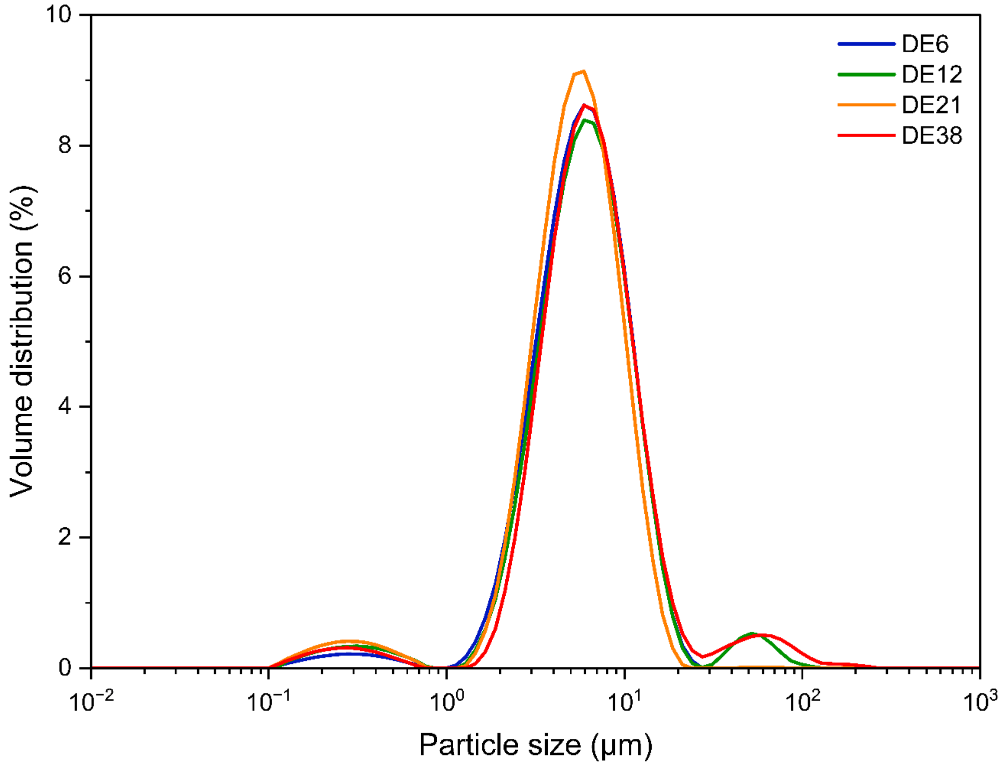

Figure S3. Volume-based size distribution of spray-dried emulsions containing maltodextrin DE6 (blue line), DE12 (green line), DE21 (orange line), and DE38 (red line).

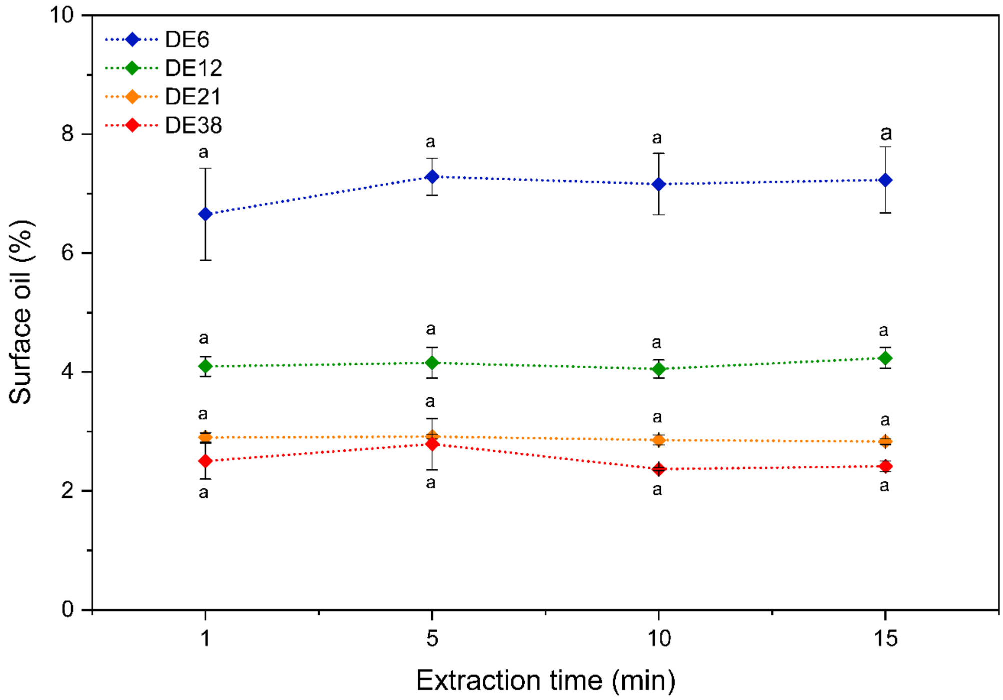

Figure S4. Mass percentage of solvent-extractable surface oil from spray-dried emulsions containing maltodextrin DE6 (blue diamonds), DE12 (green diamonds), DE21 (orange diamonds), and DE38 (red diamonds), for different extraction times: 1, 5, 10, and 15 min. Error bars display the standard deviations, and the same letters for the same DE value do not differ significantly ($p < 0.05$). Lines were added as a visual guide.

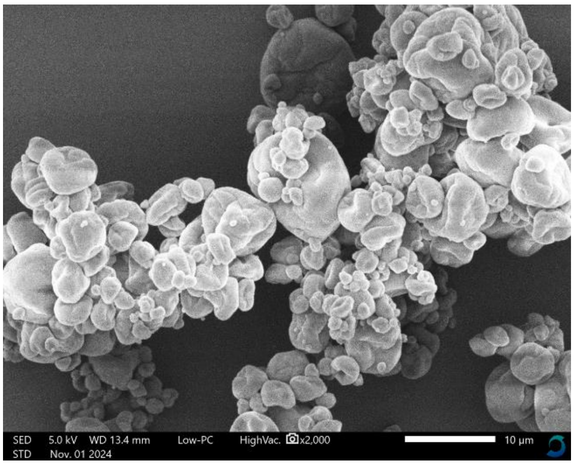

(A.i)

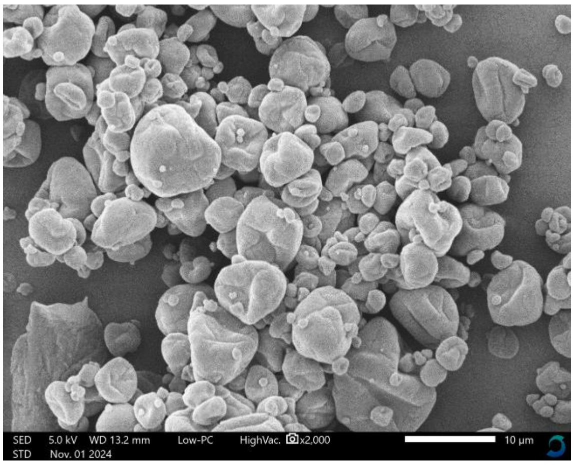

(A.ii)

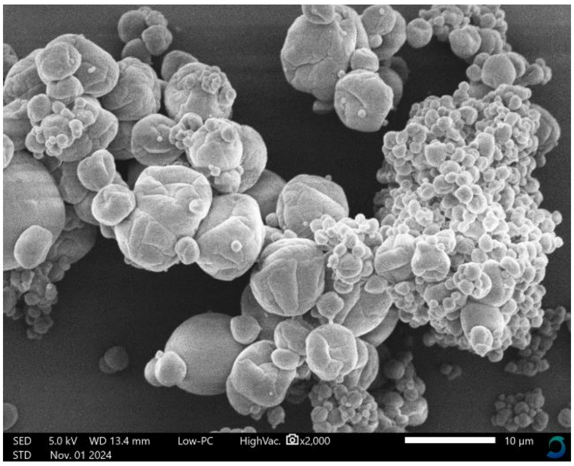

(A.i)

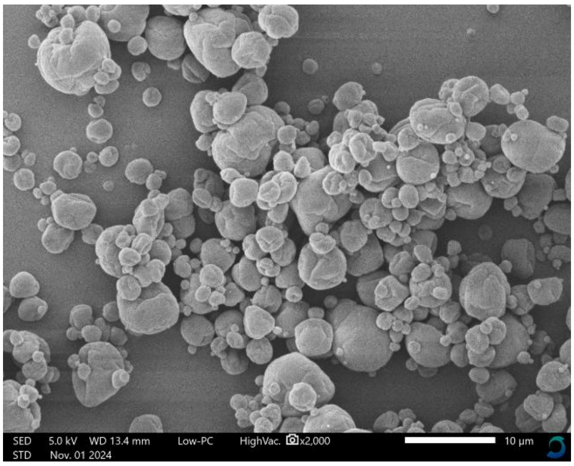

(A.ii)

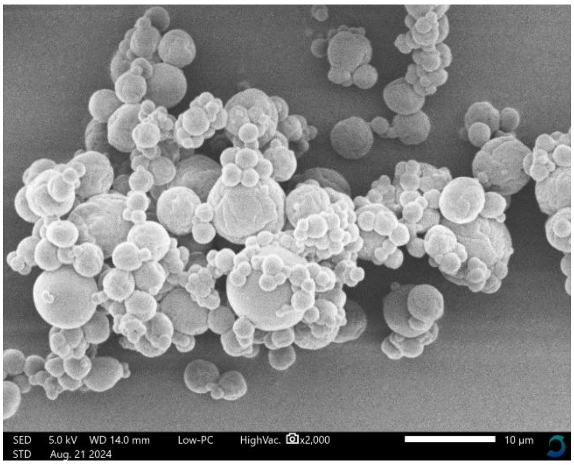

(C.i)

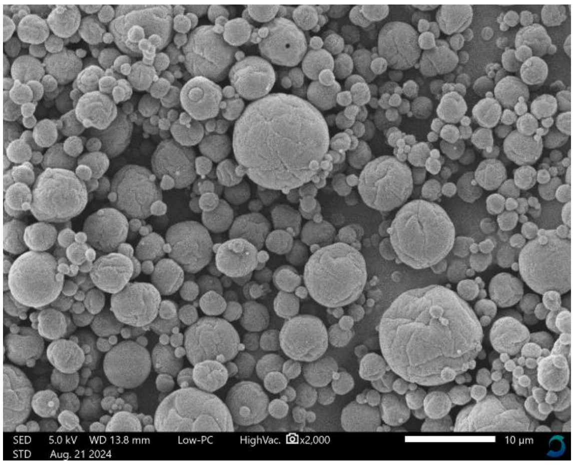

(C.ii)

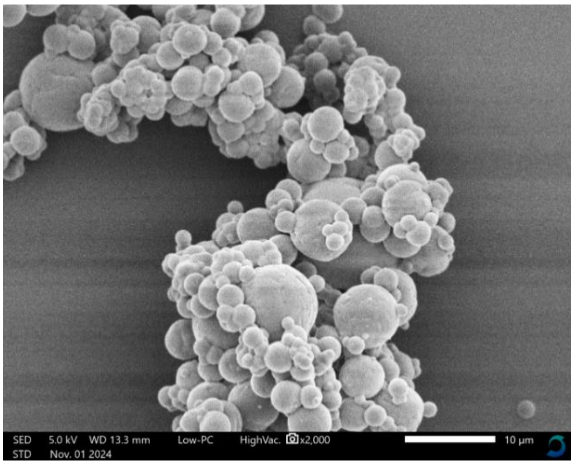

(D.i)

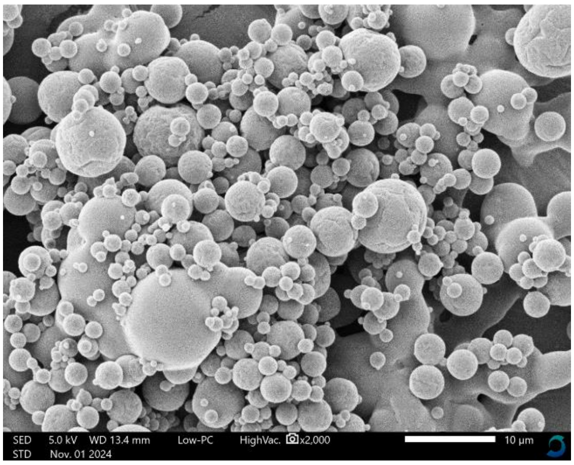

(D.ii)

Figure S5. Morphology of the spray-dried emulsions with (i) and without (ii) the surface oil, containing maltodextrin DE6 (A), DE12 (B), DE21 (C), and DE38 (D). All figures have the same magnification of 2000x, and all scale bars indicate 10 µm.

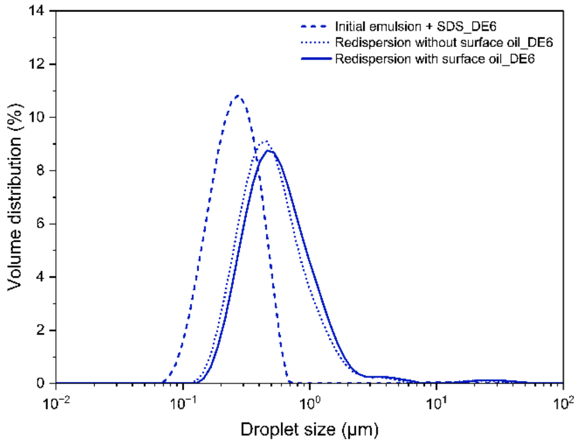

(A)

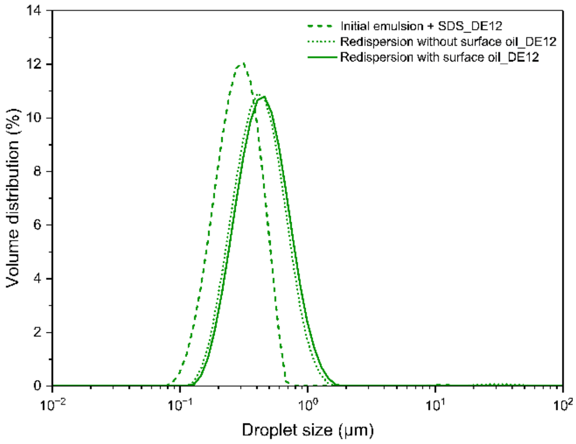

(B)

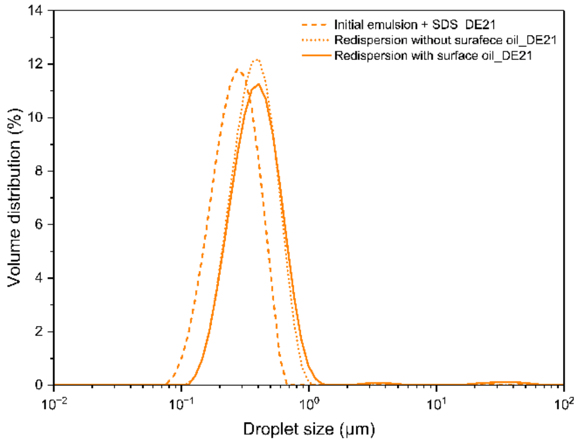

(C)

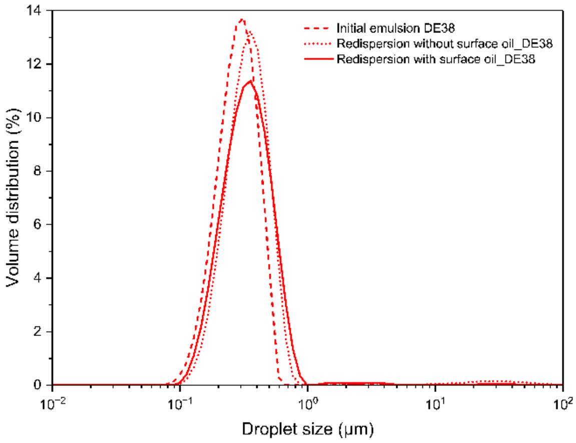

(D)

Figure S6. Volume-based size distribution of oil droplets in the initial emulsion diluted in 1:1 (v/v) of 1 wt.% SDS solution, and inside spray-dried emulsions containing maltodextrin DE6 (A), DE12 (B), DE21 (C), and DE38 (D). Dashed lines indicate the oil distribution of initial emulsions. Dotted and solid lines indicate the oil distribution of spray-dried emulsions after and before surface oil stripping, respectively.

Table S1. Other properties of spray-dried emulsions.

Sample Name	DE6	DE12	DE21	DE38
dx(10) (µm)	2.76 ± 0.2 ^{b}	2.78 ± 0.1 ^{b}	2.63 ± 0.09 ^{b}	3.10 ± 0.24 ^{a}
dx(90) (µm)	12.45 ± 0.95 ^{bc}	13.56 ± 0.37 ^{ab}	11.09 ± 0.32 ^{c}	15.80 ± 3.84 ^{a}
Span (%)	1.57 ± 0.15 ^{ab}	1.70 ± 0.03 ^{ab}	1.48 ± 0.02 ^{b}	1.90 ± 0.50 ^{a}

The same letters in the same row do not differ significantly ($p < 0.05$).

Table S2. Surface oil percentage of spray-dried emulsions, with the same lowercase letters in the same column, do not differ significantly ($p < 0.05$). The Sauter mean diameter (d[3,2]) of the oil droplet in the initial emulsion diluted 1:1 (v/v) in 1 wt.% SDS (E0+SDS), spray-dried emulsions with surface oil redispersed in 1 wt.% SDS, and spray-dried emulsions without surface oil redispersed in 1 wt.% SDS.

Sample Name	Surface oil (%)	d[3,2] (µm)			Span		
		E0+SDS	Redispersion with surface oil	Redispersion without surface oil	E0+SDS	Redispersion with surface oil	Redispersion without surface oil
DE6	6.65 ± 0.77 ^{a}	0.24 ± 0.01 ^{C}	0.51 ± 0.01 ^{A}	0.46 ± 0.01 ^{B}	1.17 ± 0.03 ^{A}	1.85 ± 0.10 ^{A}	1.85 ± 0.04 ^{A}
DE12	4.09 ± 0.17 ^{b}	0.28 ± 0.01 ^{C}	0.43 ± 0.01 ^{A}	0.40 ± 0.01 ^{B}	1.05 ± 0.05 ^{A}	1.25 ± 0.02 ^{B}	1.23 ± 0.01 ^{B}
DE21	2.90 ± 0.08 ^{c}	0.26 ± 0.01 ^{C}	0.38 ± 0.01 ^{A}	0.36 ± 0.01 ^{B}	1.07 ± 0.08 ^{A}	1.18 ± 0.02 ^{C}	1.06 ± 0.01 ^{C}
DE38	2.50 ± 0.30 ^{c}	0.28 ± 0.02 ^{C}	0.32 ± 0.01 ^{B}	0.34 ± 0.01 ^{A}	0.92 ± 0.10 ^{A}	1.14 ± 0.03 ^{C}	1.01 ± 0.16 ^{C}

The same uppercase letters in the same row do not differ significantly ($p < 0.05$).

Table S3. Mass percentage of d-limonene retained after the emulsification process, which is calculated by dividing the mass of d-limonene extracted after emulsification by the mass of d-limonene initially added to the emulsion and multiplied by 100%.

Sample Name	Mass percentage of d-limonene retained after emulsification (%)
DE6	97.92 ± 0.61 ^{a}
DE12	92.48 ± 2.52 ^{b}
DE21	96.54 ± 0.92 ^{a}
DE38	98.30 ± 2.62 ^{as}

The same letters in the same column do not differ significantly ($p < 0.05$).

Table S4. Mass percentage of encapsulated oil, total d-limonene retention, and d-limonene loss from surface oil and encapsulated oil of spray-dried emulsions.

Sample Name	Encapsulated oil (%)	d-limonene retention after spray drying (%)	Source of d-limonene loss	
			Surface oil* (%)	Encapsulated oil** (%)
DE6	93.35 ± 0.77 ^{a}	57.59 ± 2.42 ^{c}	6.65 ± 0.77 ^{a}	35.76 ± 0.77 ^{a}
DE12	95.91 ± 0.17 ^{b}	74.51 ± 1.24 ^{b}	4.09 ± 0.17 ^{b}	21.40 ± 0.17 ^{b}
DE21	97.10 ± 0.08 ^{c}	85.93 ± 1.01 ^{a}	2.90 ± 0.08 ^{c}	11.17 ± 0.08 ^{c}
DE38	97.50 ± 0.30 ^{c}	84.85 ± 1.38 ^{a}	2.50 ± 0.30 ^{d}	12.64 ± 0.30 ^{d}

The same letters in the same column do not differ significantly ($p < 0.05$).
** the values are the same as surface oil because no d-limonene is detected from the surface oil, indicating that all d-limonene on the surface oil is lost.*
*** the values are determined by subtracting the d-limonene lost through surface oil from the total d-limonene loss measured during spray drying.*
